# Changes in the magnitude of social inequality in the uptake of cervical cancer screening in Taiwan, a country implementing a population-based organized screening program

**DOI:** 10.1186/1475-9276-13-4

**Published:** 2014-01-09

**Authors:** Shu-Ti Chiou, Chien-Yuan Wu, Baai-Shyun Hurng, Tsung-Hsueh Lu

**Affiliations:** 1 Health Promotion Administration, Ministry of Health and Welfare, Taipei, Taiwan; 2 Institute of Public Health, National Yang-Ming University, Taipei, Taiwan; 3 Institute of Public Health, National Cheng Kung University, Tainan, Taiwan

**Keywords:** Mass screening, National Health Interview Survey, Socio-economic factors, Uterine cervical neoplasm

## Abstract

**Introduction:**

We sought to examine changes in the magnitude of social inequality in the uptake of cervical cancer screening between 2001 and 2009 in Taiwan.

**Methods:**

We used data from the 2001 and 2009 Taiwan National Health Interview Surveys to calculate the absolute (slope of index of inequality, SII) and relative (relative index of inequality, RII) summary measures of social inequality in the uptake of Pap smear tests to indicate the magnitude of social inequality.

**Results:**

The prevalence of having had a Pap smear during the previous 3 years increased in each age and socioeconomic group from 2001 to 2009. The SII and RII by urbanization and education level decreased significantly, while the SII and RII by income level increased significantly between the two study years. The largest increase in inequality of prevalence from 2001 to 2009 was between women living in suburban and rural areas with highest income level and women live in metropolitan areas with lowest income level.

**Conclusions:**

The changes in magnitude of social inequality in the uptake of cervical cancer screening differed by indicators of socioeconomic position. Further studies are needed to explore the mechanisms that result in social inequality by different indicators of socioeconomic position.

## Introduction

Studies using nationally-representative survey data have demonstrated prominent social inequality in the reported use of cervical cancer screening [1-13]. In most countries, women in a higher socioeconomic position have higher rates of Pap smear test uptake than their counterparts in a lower socioeconomic position. However, according to a review, very few studies directly compare the trends in socioeconomic inequality in the uptake of cervical cancer screening [14]. An international comparison study further indicated that the magnitude of social inequality in the prevalence of the uptake of cervical cancer screening is lower in countries implementing a population-based organized screening program [11]. Nevertheless, little is known regarding the changes in the magnitude of social inequality in a country implementing population-based organized screening program in an Asian country. In this study, we examine changes in the magnitude of social inequality in the reported uptake of cervical cancer screening between 2001 and 2009 in Taiwan, a country that has been implementing population-based organized screening program since 1995.

## Methods

### Organized screening program in Taiwan

According to the definition proposed by Miles et al., the cervical screening program in Taiwan is an organized system [15]. The National Cervical Cancer Screening Registry was established in 1995 to help public health nurses to identify women who should be invited for a Pap smear and to monitor whether any screening-detected abnormalities have been followed-up and treated [16,17]. However, one limitation of the National Cervical Cancer Screening Registry is that there is no personal information on socioeconomic position; therefore we have to use the National Health Interview Survey datasets to examine the relationship between socioeconomic position and prevalence of Pap smear usage.

### National Health Interview Survey in Taiwan

The National Health Interview Survey is a nationally-representative survey of the total population of Taiwan conducted by the Bureau of Health Promotion every four years. A multistage stratified systematic sampling design following the principle of probability proportional to size was applied in both the 2001 and 2009 surveys. Data were collected by face-to-face interview [18]. The response rate was 93.8% (25,464/27,160) in the 2001 survey and 84.0% (25,636/30,528) in the 2009 survey. One possible explanation of the difference in response rate between 2001 and 2009 is that the unit of sampling in 2001 was by household and in 2009 it was by individual person.

### Variables

The dependent variable was having had a Pap smear during the previous 3 years, which was determined by the question: “In what year and month did you have a Pap smear, if ever?” The independent variables included age and three indicators of the respondent’s socioeconomic position: residential urbanization level, educational level, and monthly household income. The definitions of variables are illustrated in Table 1.

**Table 1:** Definitions of variables

**Variable**	**Definition**
Dependent variable	
Pap smear	If the respondent received Pap smear in the previous 3 years;
	yes = 1, otherwise = 0
Independent variables	
Age	
30-39	If the respondent’s age is in 30–39 years; yes = 1, otherwise = 0 (reference group)
40-49	If the respondent’s age is in 40–49 years; yes = 1, otherwise = 0
50-59	If the respondent’s age is in 50–59 years; yes = 1, otherwise = 0
60-69	If the respondent’s age is in 60–89 years; yes = 1, otherwise = 0
Urbanization level	
Metropolitan	If the respondent’s resident place is in Taipei City or Kaohsiung City; yes = 1, otherwise = 0 (reference group)
Urban	If the respondent’s resident place is in Cities other than Taipei City or Kaohsiung City; yes = 1, otherwise = 0
Suburban	If the respondent’s resident place is in “Zhen” (township); yes = 1, otherwise = 0
Rural	If the respondent’s resident place is in “Xiang” (village); yes = 1, otherwise = 0
Educational level	
Primary or lower	If the respondent’s highest education level is primary school or lower; yes = 1, otherwise = 0 (reference group)
Secondary	If the respondent’s highest education level is junior high school; yes = 1, otherwise = 0
High school	If the respondent’s highest education level is senior high school; yes = 1, otherwise = 0
College or university	If the respondent’s highest education level is college or university; yes = 1, otherwise = 0
Graduate school	If the respondent’s highest education level is graduate school; yes = 1, otherwise = 0
Household monthly income (NT dollars)	
≤29,999	If the respondent’s reported income is ≤29,999; yes = 1, otherwise = 0 (reference group)
30,000–49,999	If the respondent’s reported income is 30,000–49,999; yes = 1, otherwise = 0
50,000–69,999	If the respondent’s reported income is 50,000–69,999; yes = 1, otherwise = 0
70,000–99,999	If the respondent’s reported income is 70,000–99,999; yes = 1, otherwise = 0
≥100,000	If the respondent’s reported income is ≥100,000; yes = 1, otherwise = 0

### Analysis

The prevalence of having had a Pap smear in the previous 3 years was calculated by age and three indicators of socioeconomic position. We then examined the correlations (Cramer’s V coefficient) among variables, and used the variation inflation factor (VIF) to address the concern of colinearity between covariates. The adjusted odds ratio (aOR) of having had a Pap smear in the previous 3 years for women in each socioeconomic group compared with women in a reference group was computed according to multivariate logistic regression. We also examined the interactions between different independent variables in predicting the outcome. For variables with significant interaction effects, we further stratified the variables and computed the aOR for stratified categories.

### Measure of inequality

Because we used indicators of socioeconomic position with hierarchical order (urbanization, education and income level), we were able to use regression-based measures of social inequality [19]. The slope index of inequality (SII) is the linear regression coefficient which represents the relation between the frequencies of health behavior (i.e., undertaking a Pap smear in this study) in each socioeconomic category and the hierarchical ranking of each category on the social scale [20]. The SII can be interpreted as the absolute change in frequency of health behavior when one goes from the lowest level in the social hierarchy to the highest level.

Because SII is an absolute measure, it is sensitive to changes in the mean frequency of health behaviors of population. If the mean frequency of health behavior increases in the same proportion in all the socioeconomic categories, the SII will increase, whereas the relative differences remain constant. One alterative that has been proposed is the relative index of inequality (RII), which can be estimated by dividing the predicted value of the regression at the highest point by the predicted value of the regression at the next highest point. The RII is frequently calculated by logistic regression at the lowest point. After the logistic transformation of the dependent variable, the exponent of the regression coefficient represents the RII [20].

Here is a simple example to help readers better understand the implication of SII and RII. Suppose the prevalence of taking Pap smear for low, middle, and high socioeconomic positions were 60%, 65% and 70%, respectively. In this example, there was a 5% point of increase in prevalence from low socioeconomic position to the adjacent higher socioeconomic position, the SII would be around 0.08 (5/60) and the RII would be around 1.08 (65/60 or 70/65).

As the main concern of this study was to examine changes in the magnitude of social inequality between 2001 and 2009, we pooled data from both years and included an interaction term between SII, RII, aOR and survey year in the model.

As Martens pointed out, the use of relative rates, relative risks, or odds ratios can actually be detrimental to furthering political actions. In the realm of policy, showing the rates by socioeconomic group on absolute differences may be better understood intuitively [21]. Thus, we presented changes in both rate ratio and rate difference between 2001 and 2009 by three indicators socioeconomic position to see if there were different implications for policy decision makers.

## Results

Women aged 30–69 years who had not had a hysterectomy were included in this study, with a total of 5,704 women for the year 2001 and 6,420 women in 2009. Table 2 presents the demographic and socioeconomic characteristics of the respondents. A higher percentage of women aged 50–59 years was noted in 2009 compared with those in 2001 (25% vs. 19%). The distributions of respondents by urbanization level and household income in 2001 were similar to those in 2009. However, fewer respondents reported monthly income in 2009. We also found that the 2009 respondents had higher education levels.

**Table 2:** Demographic and socioeconomic characteristics of women respondents, 2001 and 2009 Taiwan National Interview Survey

	**2001**		**2009**	
	***N***	***%***	***N***	***%***
Total	5,704	100.0	6,420	100.0
Age group (years)				
30–39	1,930	33.8	2,035	31.7
40–49	1,819	31.9	1,911	29.8
50–59	1,107	19.4	1,585	24.7
60–69	848	14.9	889	13.8
*Missing*	-	-	-	-
Urbanization level				
Metropolitan	1,540	27.0	2,032	31.7
Urban	1,446	25.4	1,635	25.5
Suburban	992	17.4	1,130	17.6
Rural	1,642	28.8	1,619	25.2
* Missing*	84	1.5	4	0.1
Educational level				
Primary or lower	2,393	42.0	1,681	26.2
Secondary	909	15.9	945	14.7
High school	1,523	26.7	2,069	32.2
College or university	816	14.3	1,492	23.2
Graduate school	60	1.1	225	3.5
* Missing*	3	0.1	8	0.1
Household monthly income (NT dollars)				
≤29,999	1,237 1,237	21.7	1,494	23.3
30,000–49,999	1,311 1,311	23.0	1,366	21.3
50,000–69,999	1,190 1,190	20.9	1,035	16.1
70,000–99,999	988 988	17.3	828	12.9
≥100,000	907 907	15.9	859	13.4
Missing	71 71	1.2	838	13.1

The overall prevalence of undertaking a Pap smear was 62% in 2001 and increased to 69% in 2009 (Table 3). The increase in prevalence was different between age group and was more prominent for elder woman; thus in the groups of 50–59 and 60–69 year-olds, the increase between years was by 10%. The increase of prevalence between 2001 and 2009 was less prominent in women living in metropolitan areas (an increase from 63% to 67%), compared with those in suburban areas (an increase from 63% to 71%), and rural areas (from 63% to 72%). Similarly, women with highest education level and highest household income group showed lowest magnitude of increase in prevalence between the two years (from 66% to 69% for the highest education level and from 68% to 75% for the highest income group). Noteworthy women with university or graduate education level had lower prevalence rate (69% in 2009) than those with high school education level (73%).

**Table 3:** Number and prevalence (%) of women who underwent a Pap smear in the previous 3 years by age and socioeconomic position, 2001 and 2009 Taiwan National Interview Survey

	**2001**		**2009**		***p-value***
	***n***	***%***	***n***	***%***	
Total	3,469	62.4	4,415	69.2	<.0001
Age group (years)					
30-39	1155	63.8	1387	68.4	0.0027
40-49	1241	68.8	1404	73.8	0.0008
50-59	666	60.5	1114	70.9	<.0001
60-69	407	48.4	510	58.3	<.0001
Urbanization level					
Metropolitan	953	63.1	1353	67.1	0.0118
Urban	853	60.8	1109	68.1	<.0001
Suburban	608	63.0	800	71.3	<.0001
Rural	1000	62.8	1150	71.5	<.0001
Education level					
Primary or lower	1315	55.5	1051	63.4	<.0001
Secondary	600	67.4	669	71.2	0.0817
High school	1008	68.8	1508	73.2	0.0047
College or university	545	66.0	1184	69.2	0.1030
Household monthly income (NT dollars)					
<=29,999	676	56.0	936	63.5	<.0001
30,000-49,999	796	62.2	933	68.5	0.0007
50,000-69,999	750	64.8	751	72.9	<.0001
70,000-99,999	607	63.4	656	79.3	<.0001
> = 100,000	601	68.1	647	75.4	0.0007

Table 4 shows correlation matrix among variables in 2001 and 2009. Although the four independent variables were significantly correlated with each other, the VIF for all variables are less than 2, so the threat of colinearity between variables was not so significant (data not shown). We therefore put all variables into the final multivariate logistic regression model. A significant interaction term between urbanization and income were noted, we thus stratified urbanization and income into five categories in the final multivariate regression model.

**Table 4:** Correlations (Cramer’s V Coefficient) among variables related to the uptake of Pap smear in Taiwan National Interview Survey

	**Uptake_2001**	**Age_2001**	**Urbanization_2001**	**Education_2001**	**Income_2001**
Uptake_2001	--	0.1378***	0.0199	0.1264***	0.0817***
Age_2001		--	0.0551***	0.3430***	0.1146***
Urbanization_2001			--	0.1761***	0.1270***
Education_2001				--	0.2052***
Income_2001					--
		Age_2009	Urbanization_2009	Education_2009	Income_2009
Uptake_2009	--	0.1052***	0.0420*	0.0821***	0.1214***
Age_2009		--	0.0391**	0.3841***	0.1703***
Urbanization_2009			--	0.1203***	0.1121***
Education_2009				--	0.3061***
Income_2009					--

***p value < .0001.

**p value < .001.

*p value < .05.

The results of final multivariate logistic regression model are presented in Table 5. The SII and RII by age moderately increased from 2001 to 2009, and the main increase occurred in women aged 50 years and above. The SII and RII by urbanization level were, respectively, 0.18 (95% CI 0.01-0.27) and 1.20 (95% CI 1.10-1.31) in 2001, and significantly decreased to 0.13 (95% CI 0.04-0.21) and 1.14 (95% CI 1.04-1.24) in 2009 according to the examination of interaction term between urbanization and year. People living in rural areas showed the most prominent decrease in magnitude of inequality, as shown by the aOR which was 1.87 (95% CI 1.38-2.54) in 2001 and decreased to 1.33 (95% CI 1.02-1.74).

**Table 5:** Adjusted odds ratios (aOR), relative index of inequality (RII) and slope index of inequality (SII) of having had a Pap smear in the previous three years by age and socioeconomic position, 2001 and 2009 Taiwan National Health Interview Survey

	**2001**		**2009**		***p*** **value for interactions with years**
	**aOR**	**95% CI**	**aOR**	**95% CI**	
Age group (years)					
30-39	1.00		1.00		
40-49	1.46	(1.26–1.70)	1.33	(1.14–1.55)	<.0001
50–59	1.15	(0.95–1.38)	1.32	(1.10–1.59)	0.1413
60–69	0.79	(0.64–0.97)	0.83	(0.66–1.05)	0.0272
SII	-0.08	(-0.14––0.01)	0.01	(-0.07–0.08)	0.0257
RII	0.93	(0.87–0.99)	1.01	(0.94–1.08)	0.0257
Urbanization level					
Metropolitan	1.00		1.00		
Urban	1.23	(0.84–1.79)	0.71	(0.52–0.98)	0.3422
Suburban	1.75	(1.27–2.42)	1.20	(0.91–1.59)	0.0185
Rural	1.87	(1.38–2.54)	1.33	(1.02–1.74)	0.0015
SII	0.18	(0.10–0.27)	0.13	(0.04–0.21)	0.0001
RII	1.20	(1.10–1.31)	1.14	(1.04–1.24)	0.0001
Education level					
Primary or lower	1.00		1.00		
Secondary	1.63	(1.36–1.96)	1.23	(0.99–1.51)	<.0001
High school	1.68	(1.41–1.99)	1.32	(1.09–1.61)	<.0001
College or university	1.38	(1.12–1.70)	1.00	(0.80–1.25)	0.0012
SII	0.14	(0.07–0.21)	0.00	(-0.07–0.08)	<.0001
RII	1.15	(1.08–1.23)	1.00	(0.93–1.08)	<.0001
Household monthly income (NT dollars)					
<=29,999	1.00		1.00		
30,000–49,999	1.57	(1.16–2.13)	1.08	(0.83–1.42)	0.0659
50,000–69,999	1.71	(1.25–2.32)	1.34	(1.01–1.79)	0.0130
70,000–99,999	1.65	(1.21–2.26)	2.06	(1.52–2.79)	0.0333
> = 100,000	3.06	(2.10–4.45)	1.80	(1.29–2.52)	<.0001
SII	0.15	(0.09–0.22)	0.21	(0.14–0.28)	<.0001
RII	1.16	(1.09–1.24)	1.24	(1.16–1.32)	<.0001
Urbanization*Income					
Metropolitan, <=29,999	1.00		1.00		
Urban, 30,000–99,999	0.78	(0.51–1.19)	1.53	(1.05–2.23)	0.3309
Urban, > = 100,000	0.55	(0.32–0.94)	1.32	(0.82–2.14)	0.5982
Suburban & rural, 30,000–99,999	0.68	(0.48–0.96)	1.03	(0.75–1.42)	0.2475
Suburban & rural, > = 100,000	0.31	(0.20–0.50)	1.03	(0.64–1.65)	0.0005

Urbanization*study year means interaction term.

With regard to education level, SII and RII were, respectively, 0.14 (95% CI 0.07-0.21) and 1.15 (95% CI 1.08-1.23) in 2001; and significantly decreased to <0.001 (95% CI -0.07-0.08) and 1.00 (95% CI 0.93-1.08) in 2009. The magnitude of decrease in aOR was most prominent among women with a secondary educational level (from 1.63 to 1.23) and women with a high school education level (from 1.68 to 1.32).

In terms of household monthly income, the SII and RII were, respectively, 0.15 (95% CI 0.09-0.22) and 1.16 (95% CI 1.09-1.24) in 2001. These significantly increased to 0.21 (95% CI -0.14-0.28) and 1.24 (95% CI 1.16-1.32) in 2009. The increase in inequality was mainly confined to people with monthly income of 70,000-99,999. The aOR of this group was 1.65 in 2001 and increased 2.06 in 2009. Stratified analysis further suggested that the inequality in prevalence between women who live in suburban and rural areas with highest income level compared with women live in metropolitan areas with lowest income level showed the most significant increase in inequality between years: the aOR was 0.31 (95% CI 0.20-0.50) in 2001 and increased to 1.03 (95% CI 0.64-1.65) in 2009.

**Figure 1:**
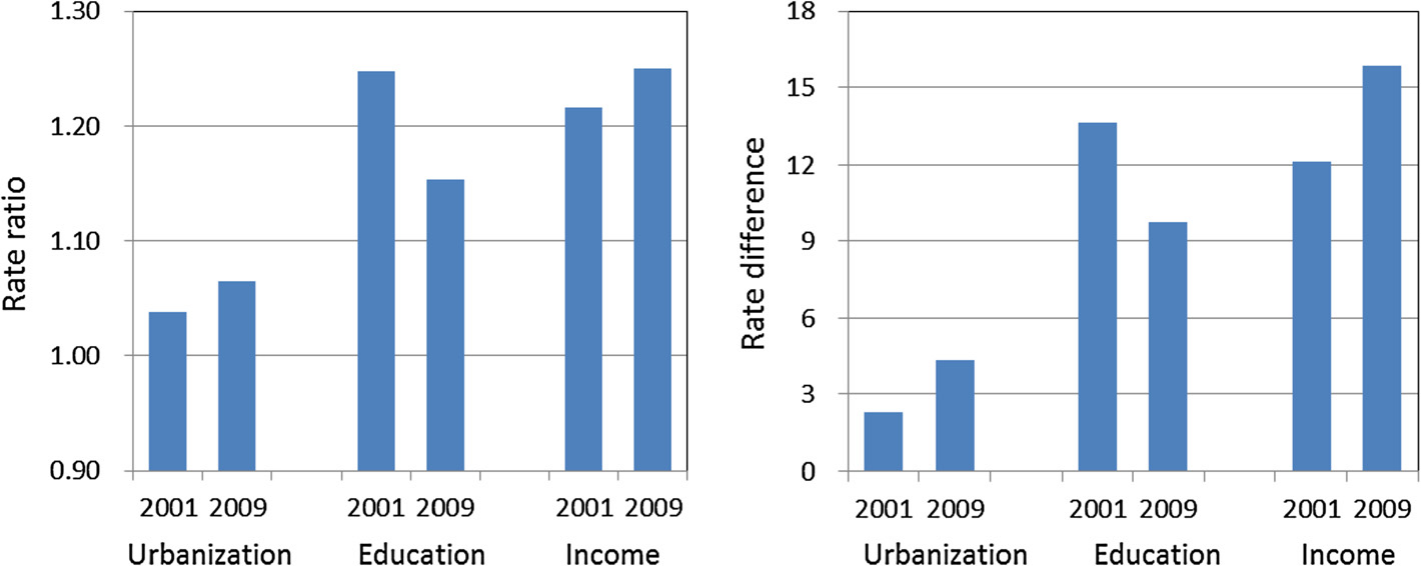
Changes in magnitude of social inequality (rate ratio versus rate difference) in the uptake of Pap smear between 2001 and 2009 according to Taiwan National Health Interview Survey.

Figure 1 illustrates changes in both rate ratio and rate difference in the uptake of Pap smear tests by the three socioeconomic variables. The pattern of change according to rate ratio and rate difference was similar.

## Discussion

The findings of this study suggest that the overall prevalence of cervical cancer screening uptake increased from 2001 to 2009. However, we observed mixed results regarding the changes in relationship between socioeconomic position and the uptake of Pap smear tests. While the magnitude of social inequality by urbanization and education level decreased, the magnitude of social inequality by income level increased. Specifically, the greatest increase of inequality between 2001 and 2009 in screening prevalence occurred between women living in suburban and rural areas with highest income level and women living in metropolitan areas with lowest income level.

A review study that examined the impact of interventions to improve attendance in female cancer screening among lower socioeconomic groups concluded that while organized population screening programs are successful in increasing overall participation rates, they may not necessarily be able to substantially reduce social inequalities [14]. We found the following two studies that specifically examined changes in the magnitude of social inequality in the uptake of cancer screening practices conducted since the publication of that review [22,23]. An organized breast cancer screening program was implemented in 2001 in Belgium. The prevalence of mammography use in women aged 50–69 years increased from 2001 to 2004; however, the RII by education level also increased [22]. In Korea, the magnitude of social inequality by education level in undertaking gastric cancer screening decreased from 2005 to 2009, but the magnitude of social inequality by income increased [23].

Our findings in Taiwan are similar to those in Korea: we found a decrease in magnitude of social inequality by education level and an increase in magnitude of social inequality by income level. The most often cited hypothesis is the “inverse equity hypothesis” proposed by Victora, which suggests that effective new interventions will initially reach those of a higher socioeconomic position and will only later filter down to those of poorer status [24]. Inequalities in coverage, morbidity, and mortality therefore increase first and then reduced later after lower socioeconomic class have goo access to the intervention. If this hypothesis applies for cervical cancer screenings in Taiwan between the years of 2001 and 2009, it implies that the availability of screening is still limited to those with greater incomes. Yet this inequality is not likely due to financial barriers because the Bureau of Health Promotion covers the fee for Pap smear examination in all medical settings under the National Health Insurance scheme (which has been in place since 1995). The findings of this study also suggest that there were interaction effects between urbanization and income level. Further studies are needed to explore the possible mechanisms resulting in the increase in magnitude of social inequality by income level.

The findings of this study further suggest that different indicators of socioeconomic position show alternate pictures of changes in magnitude of social inequality. The magnitude of social inequality as shown by urbanization level in both study years was smaller than those based on education and income level differences. One possible explanation was the launch of outreach community-based multiple disease screening program in many rural areas in some cities/counties, which resulted in the increase of prevalence cervical cancer screening [25]. The multiple disease screening program used the Pap smear screening program as a base to integrate other screening regimens encompassing four other neoplastic diseases (liver, breast, colon and oral cancer) and three chronic diseases (hypertension, hyperlipidemia, hyperglycemia). The physicians and public health nurses would outreach to many rural areas in which the screening rates were relatively low.

The decrease in magnitude of social inequality by education level was mainly due to the prominent increase in prevalence of the uptake of cervical cancer screening among women with primary or lower education level. This, again, may have been due to the launch of community-based, multiple disease screening outreach program in many rural areas. One evaluation study indicated that the outreach programs are most beneficial to elderly, widowed and less-educated women in rural areas [26]. This would also help explain why there was such a large increase of prevalence of screening in the older populations between years as well. As the demographic characteristics of respondents in 2009 were older and with higher education level than those in 2001, which would be one possible explanation of decrease in the magnitude of social inequality by educational level, as women with the highest education level have lower prevalence rate. The multiple disease screening program is a kind of intervention targeting socioeconomic disadvantages, which could effectively tackle the social inequality in health [27].

Several limitations are worthy of note. First, similar to other studies using National Health Interview Survey datasets, that there might be some recall bias in the self-reporting of the uptake of Pap smear screening. However, as the main objective of this study was to compare the social inequality in the uptake of Pap smear between 2001 and 2009, it is very unlikely that the recall bias changed markedly between the two study years. Another limitation was we could not obtain three waves of data for comparison (the interim 2005 National Health Interview Survey cervical cancer screening questions were not comparable). The third limitation was the higher non-respondent rate and missing rate in providing income information in 2009 wave, which would affect the interpretations of the results.

## Conclusion

We conclude that despite the increase in the prevalence of the uptake of cervical cancer screening between 2001 and 2009, the changes in magnitude of social inequality in the uptake of cervical cancer screening differed by indicators of socioeconomic position. Further studies are needed to explore the different mechanisms resulting in social inequality by different indicator of socioeconomic position.

## Competing interests

All authors declared no competing interests.

## Authors’ contributions

STC initiated the idea and supervised the process of study. STC and THL participated in the study design and literature review. All authors participated in the interpretation of the results and critically review the manuscript. All authors read and approved the final manuscript.
